# Npas4 impairs fear memory via phosphorylated HDAC5 induced by CGRP administration in mice

**DOI:** 10.1038/s41598-021-86556-w

**Published:** 2021-03-26

**Authors:** Narumi Hashikawa-Hobara, Shuta Mishima, Chihiro Okujima, Youdai Shitanishi, Naoya Hashikawa

**Affiliations:** Department of Life Science, Okayama University of Science, 1-1 Ridai-cho, Kita-ku, Okayama, 700-0005 Japan

**Keywords:** Fear conditioning, Epigenetics and behaviour

## Abstract

The relationships among neuropeptide, calcitonin gene-related peptide (CGRP), and memory formation remain unclear. Here, we showed that the intracerebroventricular administration of CGRP impaired the traumatic fear memories, in a widely studied animal model of post-traumatic stress disorder. We found that CGRP administration suppressed fear memory by increasing neuronal PAS domain protein 4 (Npas4), phosphorylated histone deacetylase 5 (HDAC5), and protein kinase D (PKD). We also discovered that Npas4 knockdown inhibited CGRP-mediated fear memory. CGRP decreased the binding between HDAC5 and the Npas4 enhancer site and increased the binding between acetylated histone H3 and the Npas4 enhancer site. The pharmacological inhibition or knockdown of PKD attenuated the CGRP-mediated impairment of fear memory and the increased phosphorylation of HDAC5 and Npas4 expression. Our findings demonstrated that the CGRP-PKD pathway was associated with the histone H3 acetylation-Npas4 pathway. These results suggested a novel function for CGRP on fear memory, through epigenetic regulation.

## Introduction

Calcitonin gene-related peptide (CGRP), a potent vasodilator^1^ and neurotransmitter in the central nervous system^2^, is a 37-amino-acid peptide. CGRP is distributed throughout the central and peripheral nervous systems. CGRP-containing neurons are found in the hypothalamus, preoptic area, amygdala, thalamus, hippocampus (CA3 pyramidal cells), and dentate gyrus granule cells^3,4^, and CGRP has been reported to be involved in various behaviors associated with anxiety. Intracerebroventricular (i.c.v.) CGRP infusions have been shown to evoke anxiety behaviors^5^ and to improve depression-like behaviors^6^. However, the role played by CGRP memory processes is less well-understood.

Memory can be divided into sensory memory, short-term (or working) memory, and long-term memory. In the present study, we investigated the effects of CGRP injections into the brain on memory formation. Spatial memory was tested using the hippocampus-dependent Morris water maze (MWM) task, whereas working memory was tested using a Y-maze test. Fear memory was tested using both a passive avoidance test and a contextual fear learning test.

CGRP may play an important role during the development of fear responses. CGRP-enhanced fear memory retention, as assessed by passive avoidance latency, was first observed by Kovács et al. in 1992^7^. CGRP was found to attenuate the learning impairments induced by dizocilpine, an N-methyl-D-aspartate (NMDA) receptor antagonist, as assessed using a step-down, passive avoidance task^8^. In contrast with these findings, more recently, Wu et al.^9^ reported that CGRP induced the extinction of fear memories in the central nucleus of the amygdala in rats. Thus, CGRP may modulate fear memory processing; however, whether CGRP enhances fear memory retention and what signaling mechanisms it acts through remain unclear.

Neuronal PAS domain protein 4 (Npas4) is a transcriptional factor that regulates the balance between excitatory and inhibitory synapses and plays an important role in gamma-aminobutyric acid (GABA)-ergic synapse development^10^. Recently, increasing evidence has suggested that Npas4 may have neuroprotective effects. Npas4 has been proposed to be a novel therapeutic target for the treatment of both depression and neurodegenerative diseases associated with synaptic dysfunction^11^. Npas4-deficient mice showed critical deficits in cognitive deficit hyperactivity and behavioral impairments^12^.

In recent years, interest has grown regarding the epigenetic regulation of gene expression, such as histone acetylation and deacetylation. One major histone deacetylase enzyme, histone deacetylase 5 (HDAC5), shuttles between the nucleus and the cytoplasm in response to extracellular signals. The phosphorylation of HDAC5 leads to its extrusion from the nucleus and has been associated with increased Npas4 gene expression^13^. Furthermore, cyclic adenosine monophosphate (cAMP) signaling induces HDAC5 nuclear import in striatal neurons^14^. However, whether and how the CGRP pathway affects HDAC5 or Npas4 expression remain unclear. In the present study, we identified a novel signaling mechanism, through which CGRP signaling increases the levels of protein kinase D (PKD), phosphorylated HDAC5, acetylated Histone H3, and Npas4 in the mouse hippocampus, and demonstrated that this regulatory process is essential for the suppression of fear memory retention.

## Results

### Intracerebroventricular CGRP injections affect fear memory

**Figure 1:**
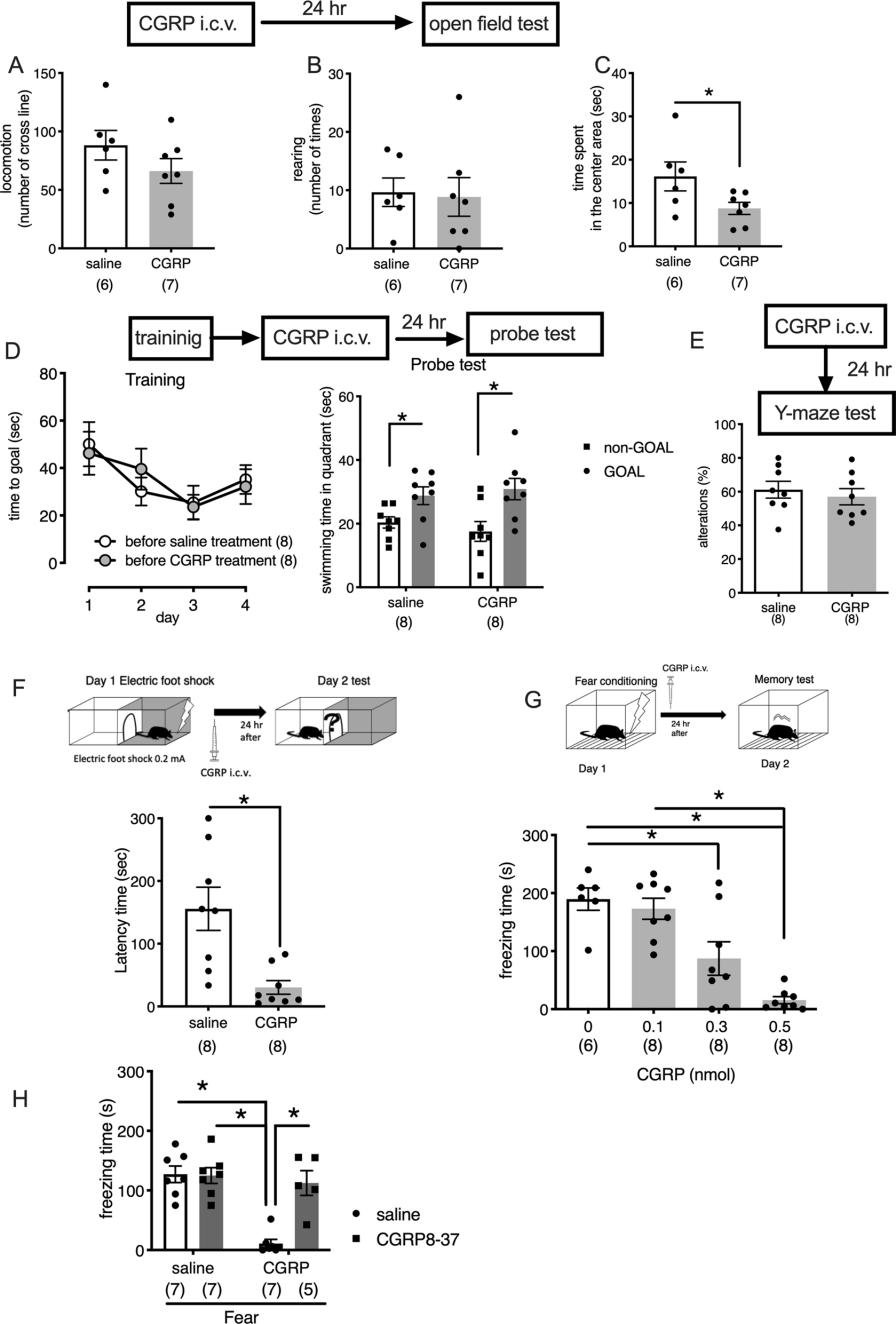
Effects of CGRP administration on general behavior and memory performance. Open field test analysis, showing locomotor activity (**A**), rearing activity (**B**) and time spent in the center area (**C**) (Welch’s t test, *p* = 0.0412), 24 h after CGRP i.c.v. (**D**) The mean escape latency in the Morris water maze task. Escape latencies were recorded during training session trials on days 1–4 (left). Changes in the percentage of time spent in the target quadrant during a 90-s testing period on day 5, 24 h after CGRP administration (right) (Welch’s t test, saline; *p* = 0.0129, CGRP; *p* = 0.0115). (**E**) CGRP effects on working memory performance in the Y maze test. (**F**) Step-through latency (in s) 24 h after foot shock (0.2 mA) (Welch’s t test, *p* = 0.0040). (**G**) The freezing time (s) during a contextual fear learning test. Fear conditioning was followed by CGRP administration (0, 0.1, 0.3, and 0.5 nmol), and freezing behavior was observed, 24 h after administration (One-way ANOVA, F_3,26_ = 16.13, with Tukey’s test). (**H**) CGRP antagonist, CGRP8-37 (0.5 nmol), blocked the CGRP (0.5 nmol)-mediated decrease in freezing time during a contextual fear learning test. CGRP8-37 was administered into the brain, simultaneously with CGRP (Two-way ANOVA, interaction, F_1,22_ = 14.3, *p* = 0.001; CGRP, F_1,22_ = 22.02, *p* = 0.0001; CGRP 8–37, F_1,22_ = 13.14, *p* = 0.0015, One-way ANOVA, F_3,24_ = 5.11, with Tukey’s test). Each bar indicates the mean ± SEM, with, significant differences shown as inserts. *p < 0.05, **p < 0.01. Numbers in parentheses indicate the animal numbers for each group. All mice were for each experiment were separate cohorts.

In the open field test, neither locomotor activity nor rearing activity was significantly affected by CGRP administration (Fig. 1A,B), although the time spent in the center area, which reflects anxiety behaviors, decreased significantly after CGRP administration (Fig. 1C, Welch’s t test, *p* = 0.0412). Next, we performed the MWM task, which can detect spatial reference memory. The platform was located 17 cm from the sidewall. Figure 1D shows the mean latencies for mice to reach the escape platform over 4 days of training. After the last training session, the mice received either CGRP or saline injections into the brain. The probe test was performed 24 h after CGRP administration. Both groups crossed the former location of the platform frequently and spent significant time in the quadrant where the platform was previously located, and CGRP administration did not affect the time spent swimming in the quadrant where the platform was located (Fig. 1D, right; Welch’s t test, saline; *p* = 0.0129, CGRP; *p* = 0.0115). We also examined working memory, using the Y-maze test, which was performed 24 h after CGRP administration; however, no significant differences were observed between the CGRP- and saline-treated groups (Fig. 1E), suggesting that CGRP did not affect spatial memory.

We next assessed whether CGRP affects fear memory in mice. In the passive avoidance test, all mice entered a dark chamber before the training foot shocks were administered. After receiving a 0.2-mA foot shock, mice received CGRP (0.5 nmol) or saline injections and were tested again, 24 h after receiving treatment. CGRP treatment significantly reduced the retention of fear memory compared with saline treatment (Fig. 1F, Welch’s t test, *p* = 0.0040). Similarly, the freezing levels during the hippocampal-dependent contextual memory task were significantly lower in the CGRP-treated mice than in the saline-treated mice, in a dose-dependent manner (Fig. 1G, One-way ANOVA, F_3,26_ = 16.13, with Tukey’s test). To clarify whether CGRP was affecting fear memory through the CGRP receptor, we administered CGRP, combined with a CGRP antagonist (CGRP8-37), which significantly blocked the CGRP-induced inhibition of fear memory retention (Fig. 1H, Two-way ANOVA, interaction, F_1,22_ = 14.3, *p* = 0.001; CGRP, F_1,22_ = 22.02, *p* = 0.0001; CGRP 8–37, F_1,22_ = 13.14, *p* = 0.0015, One-way ANOVA, F_3,24_ = 5.11, with Tukey’s test). These observations indicated that CGRP injections affected fear memory retention through the CGRP receptor.

### Npas4 plays a critical role during the CGRP-induced impairment of fear memory retention

**Figure 2:**
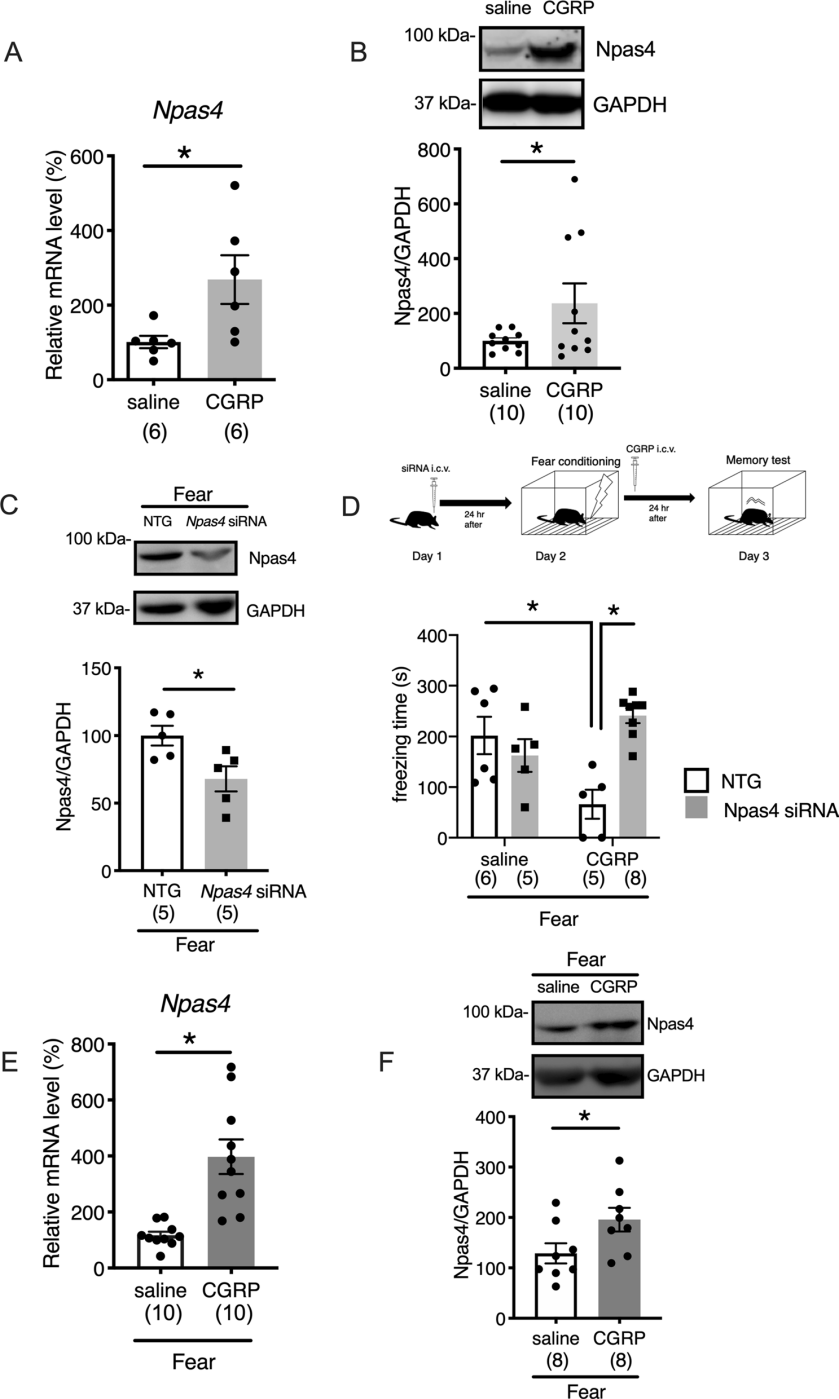
Npas4 is involved in the CGRP-mediated suppression of fear memory retention. (**A**) Npas4 mRNA (Welch’s t test, *p* = 0.0249). (**B**) Npas4 protein (Welch’s t test, *p* = 0.0473). (**C**) Npas4 protein expression with fear conditioning and Npas4 siRNA treatment (Welch’s t test, *p* = 0.0143). (**D**) Freezing time during the contextual fear learning test, after treatment with Npas4 siRNA (Two-way ANOVA, interaction, F_1,20_ = 14.79, *p* = 0.001; CGRP, F_1,20_ = 1.064, *p* = 0.3146; Npas4 siRNA, F_1,20_ = 5.976, *p* = 0.0239, One-way ANOVA, F_3,20_ = 7.375, with Tukey’s test). (**E**) Npas4 mRNA with fear conditioning (Welch’s t test, *p* = 0.0006). (**F**) Npas4 protein with fear conditioning (Welch’s t test, *p* = 0.0238). Each bar indicates the mean ± S.E.M. * *p* < 0.05. Numbers in parentheses indicate the animal numbers for each group. The full-length blots are presented in Supplementary Fig. 2.

To assess whether CGRP affects fear memory in mice, we focused on Npas4. Npas4 is a well-known transcriptional factor that is involved in neuroprotection^11^. The mRNA and protein levels of Npas4 were examined by real-time PCR and Western blotting, respectively, 24 h after the administration of CGRP or saline into the brain. Significant increases in both the *Npas4* mRNA (Fig. 2A, Welch’s t test, *p* = 0.0249) and protein expression levels (Fig. 2B, Welch’s t test, *p* = 0.0473) were observed in the mouse hippocampus following CGRP administration. Because Npas4 levels were significantly elevated by CGRP administration, we examined the effects of *Npas4* knockdown on fear memory retention in mice. Either small interfering RNA (siRNA) targeting *Npas4* or a nontargeting control siRNA was injected into the mouse brain, and after 24 h, mice received fear conditioning, followed by treatment with either CGRP or saline. To confirm that the *Npas4* knockdown resulted in reduced Npas4 protein levels in the mouse hippocampus, we measured Npas4 protein levels in mice treated with *Npas4*-siRNA or nontargeting control followed by fear conditioning. *Npas4*-siRNA treatment resulted in an approximately 33% decrease in the Npas4 level, relative to the levels observed for the nontargeting control siRNA (Fig. 2C, Welch’s t test, *p* = 0.0143). To more specifically analyze the role played by Npas4 during fear memory formation, we injected either *Npas4*-siRNA or a nontargeting control into the mouse brains and evaluated the performances of treated mice on a contextual fear learning test. The mice that received *Npas4*-siRNA combined with CGRP treatment displayed significantly increased freezing times (Fig. 2D, Two-way ANOVA, interaction, F_1,20_ = 14.79, *p* = 0.001; CGRP, F_1,20_ = 1.064, *p* = 0.3146; Npas4 siRNA, F_1,20_ = 5.976, *p* = 0.0239, One-way ANOVA, F_3,20_ = 7.375, with Tukey’s test) compared with mice treated with control siRNA combined with CGRP treatment, suggesting that Npas4 is necessary for the CGRP-induced impairment of fear memory retention. To study the possible role played by CGRP during fear conditioning, we measured the expression levels of Npas4 and phosphorylated HDAC5 in hippocampal tissue punches, after performing a contextual fear learning test. Compared with saline administration, CGRP administration produced a significant increase in the level of *Npas4* mRNA (Fig. 2E, Welch’s t test, *p* = 0.0006). As expected, the CGRP administration induced a similar increase in the Npas4 protein level (Fig. 2F, Welch’s t test, *p* = 0.0238). Npas4 has been reported to promote fear memory processes in rodents^15,16^. According to previous reports, Npas4 is rapidly induced after fear conditioning (5–30 min), returning to baseline levels within 3 or 4 h^16^. Consistent with these previous results, no significant differences were detected in Npas4 mRNA or protein levels in saline-treated animals between naïve controls and 24 h after fear conditioning (Supplemental Fig. 1A and B).

### CGRP reduces fear memory retention through the epigenetic regulation of histone H3 acetylation via HDAC5

**Figure 3:**
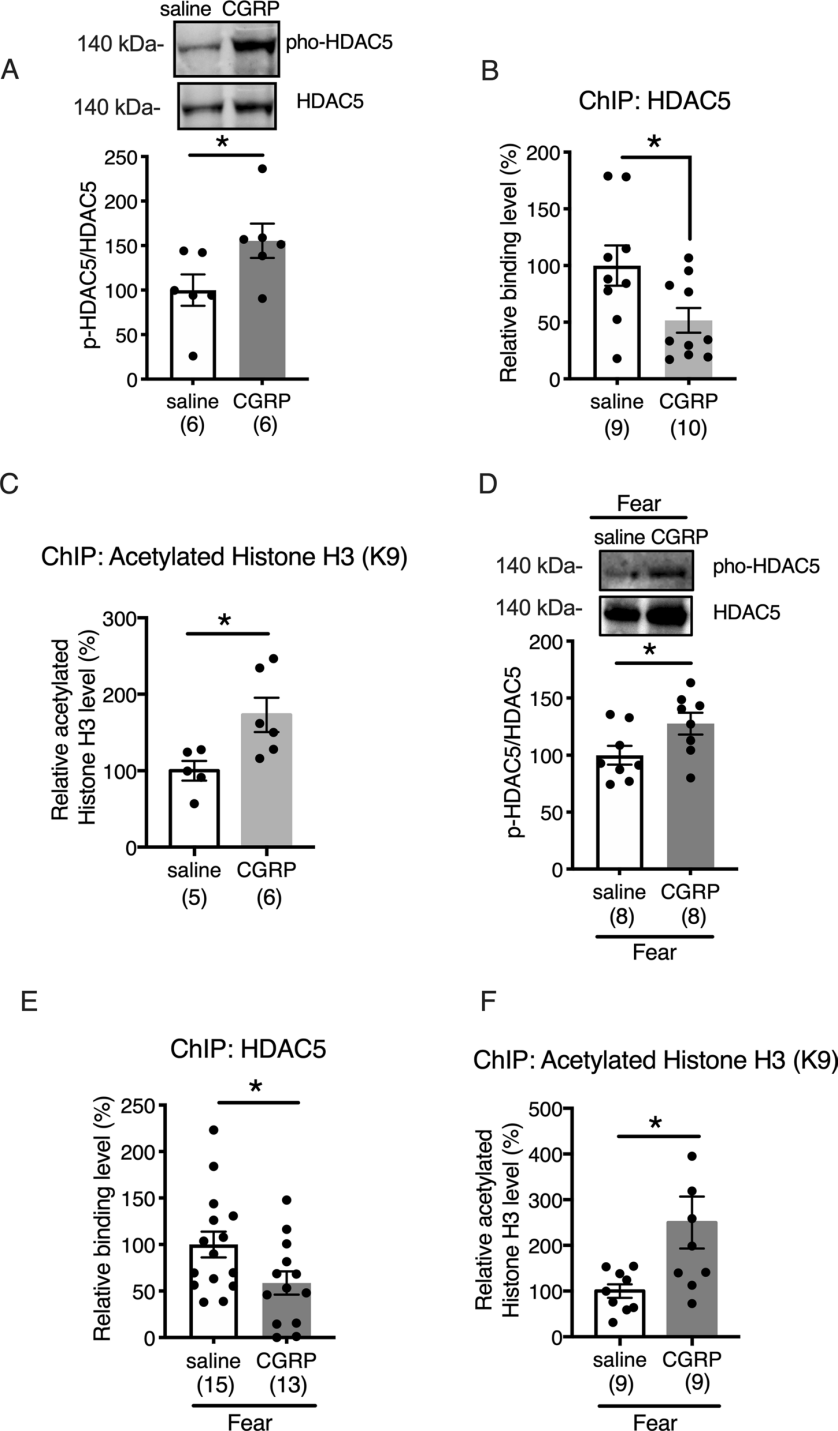
HDAC5 and histone H3 acetylation are involved in the CGRP-mediated suppression of fear memory retention. (**A**) Phosphorylated HDAC5 expression (Welch’s t test, *p* = 0.0298). (**B**) Chromatin, obtained from the mouse hippocampus, was immunoprecipitated using antibodies against HDAC5 (Welch’s t test, *p* = 0.0181) (**B**), histone H3, and acetylated histone H3 (K9) (**C**) (Welch’s t test, *p* = 0.0116). After immunoprecipitation, Npas4 enhancer expression was evaluated by qt-PCR. (**D**) Phosphorylated HDAC5 expression after fear conditioning (Welch’s t test, *p* = 0.0241). (**E**) Chromatin, obtained from the mouse hippocampus after fear conditioning, was immunoprecipitated using antibodies against HDAC5 (Welch’s t test, *p* = 0.0182) (**E**), histone H3, and acetylated histone H3 (K9) (**F**) (Welch’s t test, *p* = 0.0156). Each bar indicates the mean ± S.E.M. Student’s t-test * *p* < 0.05. Numbers in parentheses indicate the number of animals in each group. The full-length blots are presented in Supplementary Fig. 3.

Next, we focused on HDAC5 to investigate the signaling pathway associated with CGRP treatment and to determine whether CGRP is involved in the expression of Npas4. HDAC5 has been reported to bind to the enhancer region of *Npas4*, suppressing its expression; however, phosphorylated HDAC5 does not translocate to the nucleus^13^. First, we tested whether CGRP was required to phosphorylate HDAC5 in the mouse hippocampus. Compared with saline treatment, CGRP treatment significantly increased phosphorylated HDAC5 (S498) levels in the mouse hippocampus (Fig. 3A, Welch’s t test, *p* = 0.0298). Because CGRP elevated phosphorylated HDAC5 levels, we performed chromatin immunoprecipitation (ChIP) assays to determine whether HDAC5 binds to the *Npas4* enhancer site 24 h after CGRP administration. The results were expressed as a relative binding percentage, calculated as the ratio between the ChIP assay signal (bound HDAC5) and the signal for the input sample (sample without anti-HDAC5). All signals were normalized against the levels detected in the saline-treated sample, with the saline-treated level set to 100%. CGRP treatment resulted in a 50% decrease in the binding of HDAC5 to the *Npas4* enhancer site compared with saline treatment (Fig. 3B, Welch’s t test, *p* = 0.0181). Furthermore, we utilized a ChIP assay to determine whether histone H3 was acetylated in the presence of CGRP. CGRP significantly increased the levels of acetylated histone H3 bound to the *Npas4* enhancer site (Fig. 3C, Welch’s t test, *p* = 0.0116). Next, we examined whether CGRP-mediated changes in HDAC5 phosphorylation and epigenetic regulation could be observed following fear conditioning. We determined that no significant difference existed in the levels of phosphorylated HDAC5 expression in saline-treated animals between naïve controls and animals 24 h after fear conditioning (Supplemental Fig. 1C). In contrast, we found that phosphorylated HDAC5 (Ser498) was elevated in the hippocampus of CGRP-injected mice (Fig. 3D, Welch’s t test, *p* = 0.0241). Similarly, CGRP treatment combined with fear conditioning resulted in a 40% decrease in the binding of HDAC5 to the *Npas4* enhancer site, compared with saline treatment (Fig. 3E, Welch’s t test, *p* = 0.0182). Furthermore, CGRP significantly increased the level of acetylated histone H3 that was bound to the *Npas4* enhancer site (Fig. 3F, Welch’s t test, *p* = 0.0156). These data suggested that CGRP stimulates Npas4 expression by suppressing HDAC5 binding to specific *Npas4* enhancer regions, increasing histone H3 acetylation.

### The CGRP-PKD pathway is required for the impairment of fear memory retention via phosphorylated HDAC5 and Npas4

**Figure 4:**
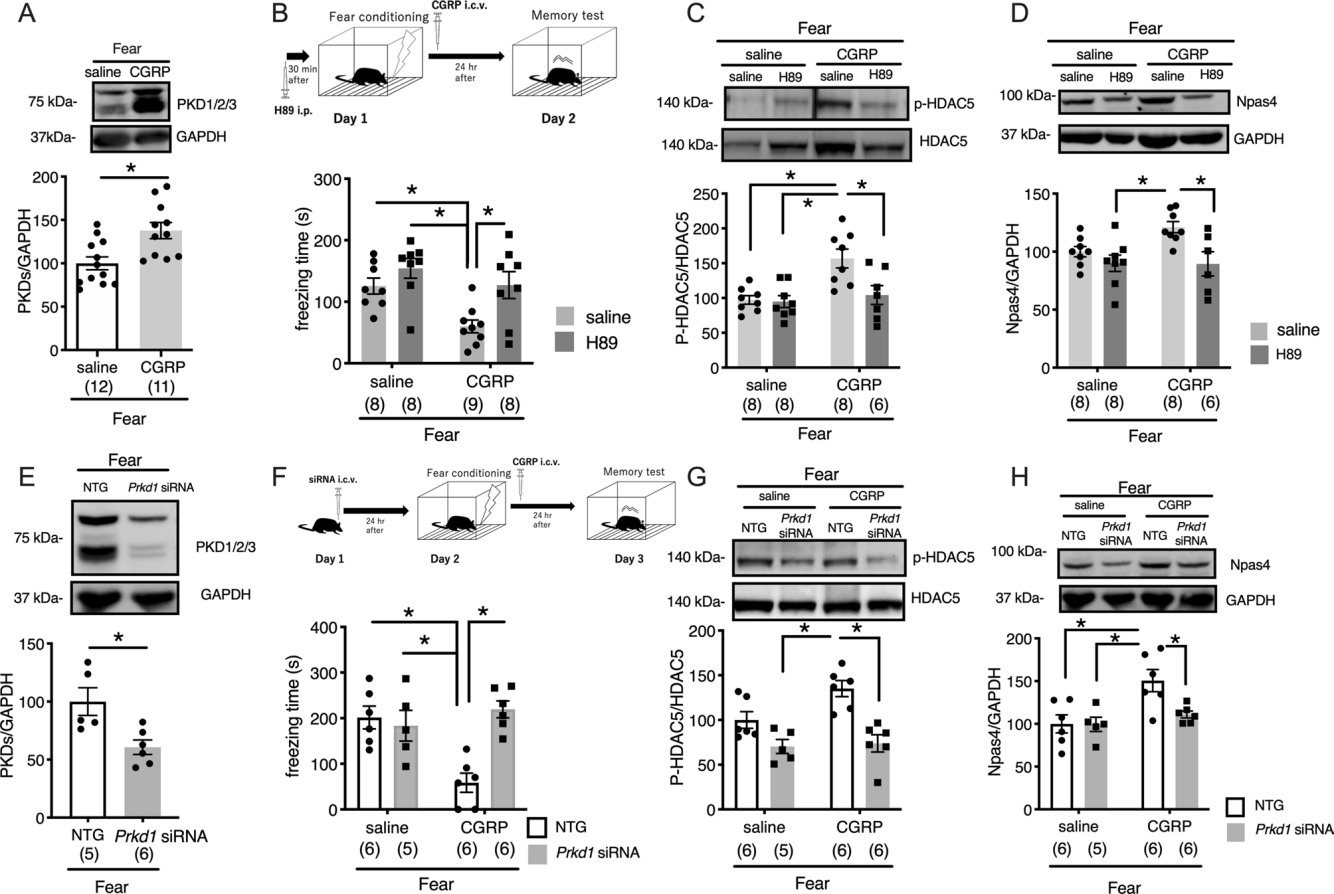
The CGRP-PKD pathway is required for the impairment of fear memory retention, via phosphorylated HDAC5 and Npas4 (**A**) PKD family member expression after fear conditioning (Welch’s t test, *p* = 0.0025). (**B**) Freezing time during the contextual fear learning test after treatment with H89 (Two-way ANOVA, interaction, F_1,29_ = 1.515, *p* = 0.2283; CGRP, F_1,29_ = 8.817, *p* = 0.0059; H89, F_1,29_ = 9.474, *p* = 0.0045, One-way ANOVA, F_3,28_ = 6.926, with Tukey’s test). (**C**) Phosphorylated HDAC5 expression (Two-way ANOVA, interaction, F_1,27_ = 5.474, *p* = 0.0269; CGRP, F_1,27_ = 10.28, *p* = 0.0034; H89, F_1,27_ = 6.535, *p* = 0.0165, One-way ANOVA, F_3,27_ = 7.663, with Tukey’s test). (**D**) Npas4 protein expression (Two-way ANOVA, interaction, F_1,26_ = 2.646, *p* = 0.1158; CGRP, F_1,26_ = 2.39, *p* = 0.1342; H89, F_1,26_ = 9.786, *p* = 0.0043, One-way ANOVA, F_3,26_ = 5.105, with Tukey’s test). (**E**) PKD family member expression after fear conditioning and Prkd1 siRNA treatment (Welch’s t test, *p* = 0.0130). (F) Freezing time during the contextual fear learning test, after treatment with Prkd1 siRNA (Two-way ANOVA, interaction, F_1,19_ = 13.36, *p* = 0.0017; CGRP, F_1,19_ = 4.814, *p* = 0.0409; Prkd1 siRNA, F_1,19_ = 8.546, *p* = 0.0087, One-way ANOVA, F_3,19_ = 9.32, with Tukey’s test). (**G**) Phosphorylated HDAC5 expression after fear conditioning and Prkd1 siRNA treatment (Two-way ANOVA, interaction, F_1,19_ = 3.005, p = 0.0992; CGRP, F_1,19_ = 4.485, *p* = 0.0476; Prkd1 siRNA, F_1,19_ = 24.79, *p* < 0.0001, One-way ANOVA, F_3,19_ = 10.86, with Tukey’s test). (**H**) Npas4 protein expression after fear conditioning and Prkd1 siRNA treatment (Two-way ANOVA, interaction, F_1,19_ = 3.992, *p* = 0.0602; CGRP, F_1,19_ = 10.22, *p* = 0.0047; Prkd1 siRNA, F_1,19_ = 4.259, *p* = 0.0530, One-way ANOVA, F_3,19_ = 6.304, with Tukey’s test). Each bar indicates the mean ± S.E.M. Student’s t-test or one-way ANOVA, with Tukey’s post hoc test. *p < 0.05. Numbers in parentheses indicate animal numbers in each group. The full-length blots are presented in Supplementary Fig. 4.

Because CGRP treatment increased Npas4 expression levels through epigenetic regulation, we examined whether the CGRP-PKD pathway was associated with the histone H3 acetylation-Npas4 pathway. The phosphorylation of HDAC5 (Ser 498) has been reported to be regulated by PKD^17^. We used antibodies to detect PKD family members (PKD1, PKD2, and PKD3). CGRP treatment significantly increased the expression levels of PKD family members (Fig. 4A, Welch’s t test, *p* = 0.0025). To investigate whether PKD plays a role in the modulation of CGRP-mediated increases in the levels of phosphorylated HDAC5 and Npas4, we administered 1 mg/kg H89, a PKD inhibitor^18,19^. H89 was injected intraperitoneally, 30 min before fear conditioning on Day 1. During the contextual fear learning test, we observed that H89 treatment significantly suppressed CGRP-mediated memory retention impairment (Fig. 4B, Two-way ANOVA, interaction, F_1,29_ = 1.515, *p* = 0.2283; CGRP, F_1,29_ = 8.817, *p* = 0.0059; H89, F_1,29_ = 9.474, *p* = 0.0045, One-way ANOVA, F_3,28_ = 6.926, with Tukey’s test). As expected, when H89 inhibited PKD activity, CGRP failed to increase phosphorylated HDAC5 levels and Npas4 protein levels (Fig. 4C, Two-way ANOVA, interaction, F_1,27_ = 5.474, *p* = 0.0269; CGRP, F_1,27_ = 10.28, *p* = 0.0034; H89, F_1,27_ = 6.535, *p* = 0.0165, One-way ANOVA, F_3,27_ = 7.663, with Tukey’s test and Fig. 4D, Two-way ANOVA, interaction, F_1,26_ = 2.646, *p* = 0.1158; CGRP, F_1,26_ = 2.39, *p* = 0.1342; H89, F_1,26_ = 9.786, *p* = 0.0043, One-way ANOVA, F_3,26_ = 5.105, with Tukey’s test).

**Figure 5:**
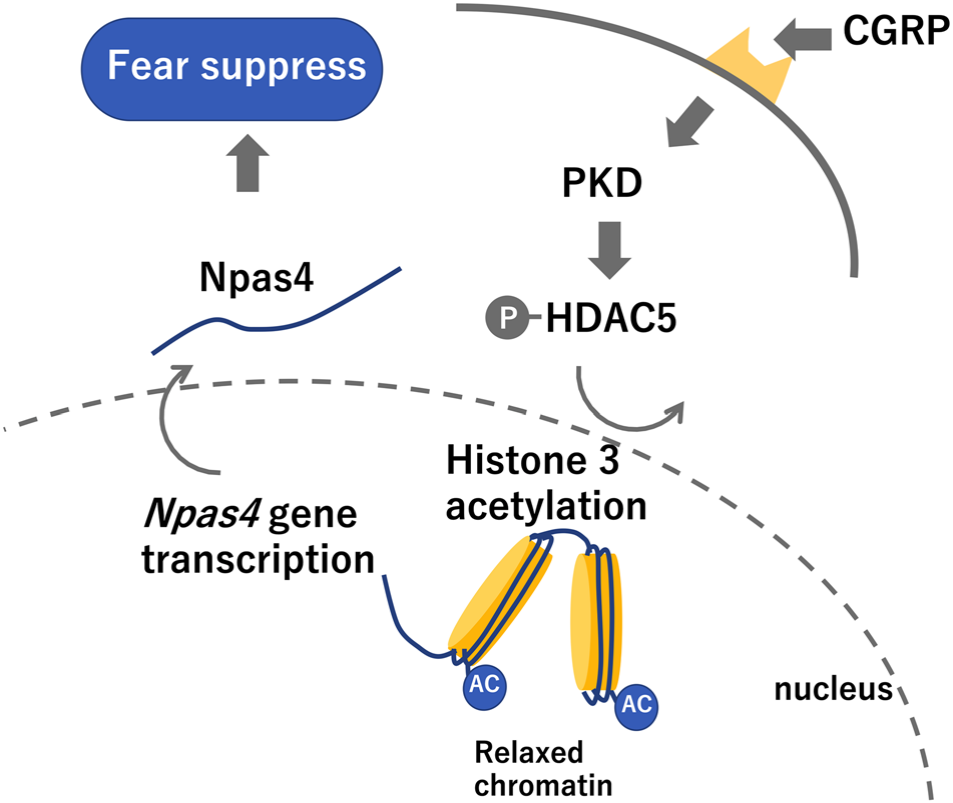
Model CGRP-induced reduction of fear memory retention through epigenetic regulation via the PKD-HDAC5-*Npas4* pathway.

H89 is widely used as a cAMP-dependent protein kinase inhibitor that affects multiple protein kinases, including protein kinase A (PKA). To determine whether PKD specifically was required for CGRP-mediated fear memory formation, we examined the effects of *PKD* knockdown on fear memory retention in mice. To confirm that the *PKD* knockdown resulted in reduced PKD expression in the mouse hippocampus, we measured PKD protein levels in mice treated with *Prkd1*-siRNA, combined with fear conditioning. *Prkd1-*siRNA treatment resulted in an approximately 40% decrease in the PKD levels (Fig. 4E, Welch’s t test, *p* = 0.0130). To more specifically analyze the role played by PKD during fear memory formation, we injected either *Prkd1-*siRNA or a nontargeting control into mouse brains and evaluated the performances of treated mice on a contextual fear learning test. Mice that received *Prkd1*-siRNA combined with CGRP treatment displayed significantly increased freezing times (Fig. 4F, Two-way ANOVA, interaction, F_1,19_ = 13.36, *p* = 0.0017; CGRP, F_1,19_ = 4.814, *p* = 0.0409; Prkd1 siRNA, F_1,19_ = 8.546, *p* = 0.0087, One-way ANOVA, F_3,19_ = 9.32, with Tukey’s test) compared with mice treated with control siRNA combined with CGRP treatment, suggesting that PKD is necessary for the CGRP-induced impairment of fear memory retention. Consistent with H89 administration, *Prkd1*-siRNA significantly reduced phosphorylated HDAC5 (Fig. 4G, Two-way ANOVA, interaction, F_1,19_ = 3.005, *p* = 0.0992; CGRP, F_1,19_ = 4.485, *p* = 0.0476; Prkd1 siRNA, F_1,19_ = 24.79, *p* < 0.0001, One-way ANOVA, F_3,19_ = 10.86, with Tukey’s test) and Npas4 protein levels (Fig. 4H, Two-way ANOVA, interaction, F_1,19_ = 3.992, *p* = 0.0602; CGRP, F_1,19_ = 10.22, *p* = 0.0047; Prkd1 siRNA, F_1,19_ = 4.259, *p* = 0.0530, One-way ANOVA, F_3,19_ = 6.304, with Tukey’s test) compared with mice treated with the nontargeting control and CGRP administration. Collectively, these data suggested that CGRP treatment, combined with fear conditioning, likely stimulates Npas4 expression by suppressing the binding between HDAC5 and specific *Npas4* enhancer regions and by increasing histone H3 acetylation, through PKD expression (Fig. 5).

## Discussion

The present study showed that CGRP suppresses fear memory retention by increasing phosphorylated HDAC5, acetylated histone H3, and Npas4 levels in the mouse hippocampus (Fig. 5). Moreover, we showed that the CGRP-PKD pathway was involved in fear memory retention and Npas4 expression. Together, these results elucidate a novel function for CGRP and a clear mechanism through which CGRP blocks fear memory retention using epigenetic regulation.

Several lines of evidence have indicated that CGRP might affect fear memory. In 1992, Kovács et al. reported that CGRP enhanced fear memory retention, as assessed by a passive avoidance latency test^7^. CGRP was also reported to attenuate learning impairments induced by the NMDA receptor antagonist dizocilpine^8^. More recently, however, Wu et al. reported the CGRP-induced extinction of fear memory in the central nucleus of the amygdala in rats^9^. Thus, several studies have reported that CGRP might affect memory formation, but the results have been controversial. Most reports have focused on the amygdala, which sends outputs to the hypothalamus and brainstem to produce fear symptoms. However, few reports have examined the relationship between CGRP and context conditioning, which serves as a model of hippocampal-dependent fear conditioning. Furthermore, few reports have examined the detailed mechanisms through which CGRP suppresses fear memory. We believe that the current study represents the first report to determine the mechanism underlying the CGRP effects on fear memory retention, which appears to involve epigenetic regulation.

*Npas4* is regulated by HDAC5, which binds to the enhancer region upstream of *Npas4* to suppress expression^13^. HDAC5 is normally phosphorylated and exists in the cytoplasm. Cocaine stimulation dephosphorylates HDAC5, causing it to translocate to the nucleus, where it attenuates the expression of target genes, including *Npas4*^13^. We demonstrated that CGRP significantly increased the residual serine phosphorylation level at S498 in HDAC5 and elevated *Npas4* expression levels in the mouse hippocampus (Figs. 2B, 3A). To clarify the relationship between the CGRP signaling cascade and phosphorylated HDAC5, we examined changes in phosphorylated HDAC5 levels following treatments using pharmacological inhibition or PKD knockdown. Mice were treated with the PKD inhibitor H89 or *Prkd1* siRNA, and the level of phosphorylated HDAC5 significantly decreased (Fig. 4C,G). Numerous studies have reported that CGRP is a G protein-coupled receptor (GPCR) that activates a major intracellular signaling cascade, resulting in increased intracellular cAMP concentrations and the downstream activation of PKA^20,21^. However, CGRP could affect not only PKA but also protein kinase C (PKC). Several studies have reported that the activation of PKC causes CGRP-mediated hyperalgesia or desensitization^20,22^. A more recent study also reported that CGRP induced the rapid and sustained activation of PKC in the cytosol^23^. Furthermore, CGRP induces the activation of calcium/calmodulin-dependent protein kinase II (CaMKII) and cAMP response element-binding protein (CREB) during neuronal signaling^24^, and recent studies have identified several protein kinases associated with CGRP-mediated effects, including CaMK and PKD family members. PKD is a serine/threonine kinase with three family members, PKD1, PKD2, and PKD3^25,26^. PKD1, formerly known as PKCµ, is the most well-studied member of this new protein kinase family within the PKD group^25^. PKD2 has been reported to be involved in tumor-promoting processes^27^. PKD3 demonstrated pro-oncogenic properties in prostate and skin cancer^28^. CaMKII, which belongs to a family of cytosolic serine/threonine protein kinases, can phosphorylate type II HDACs^29^. A recent study revealed that PKD knockdown suppressed the phosphorylation of HDAC5 S632/S498 in intestinal epithelial cells^17^. Consistent with previous reports, the results from the current study suggested that CGRP-stimulates PKD expression and increases the S498 phosphorylation of HDAC5 in the mouse hippocampus. We identified a novel role for CGRP, which mediates the phosphorylation of HDAC5 via PKD activation, resulting in the elevation of *Npas4* expression and decreased fear memory retention.

*Npas4* is a well-known immediate-early gene, which is induced rapidly when neurons are activated by membrane depolarization, seizures, or sensory signals^30,31^. Similarly, CGRP is a single polypeptide of 37 amino acids and has a short half-life (t_1/2_ 5 min) in human plasma^32^. How does a single injection of CGRP increase the levels of *Npas4* 24 h later, as observed in the present study? In the current study, we observed that the CGRP administration suppressed HDAC5 binding with *Npas4* enhancer regions, as assessed by the ChIP assay, indicating that CGRP might control epigenetic gene regulation through histone acetylation. Previous analyses of histone acetyltransferase and HDAC activities have produced long-lasting behavioral effects during conditioned place preference tests^33^. In addition, the immediate infusion of DNA methyltransferase (DNMT) inhibitors after contextual fear conditioning impaired the consolidation of long-term memory, as assessed by freezing behavior 24 h later^34,35^. Together, these studies indicated that the epigenetic control of gene expression has long-lasting effects, compared with the effects of transcriptional regulators. Furthermore, our present study demonstrated that the observed decrease in freezing time during the contextual fear learning test was significantly inhibited by *Npas4* knockdown. These results suggested that long-lasting, high levels of Npas4 are involved in the suppression of fear memory retention. Npas4 has neuroprotective effects against cerebral ischemia, neurodegeneration, and neuroinflammation^36–38^.

Npas4 dysfunction has been suggested to be involved in autism, bipolar disorder, and cognitive disorders^39–41^. Growing evidence has suggested that Npas4 stimulates the transcription of brain-derived neurotrophic factor (BDNF)^10,42,43^, which is a key mediator of synaptic plasticity in the brain. BDNF has been reported to enhance fear memory extinction^44–46^. Consistent with previous reports, we found that the CGRP-Npas4 pathway inhibited the freezing time in a contextual fear memory test by affecting epigenetic regulation. In contrast, previous studies have reported that Npas4 deficiency in mice suppressed the promotion of fear memory processes^15,16^. At present, we do not have a clear explanation for this discrepancy; however, the global conditions in knockout mice may result in the alteration of other transcription factors or neurotrophic factors, leading to different results.

To our knowledge, this study represents the first report to examine the effects of exogenous CGRP injections on fear memory retention in associating with changes in epigenetic regulation. However, this study also has some limitations. First, although we evaluated hippocampal gene expression and selected a hippocampus-dependent fear memory test, we are unable to exclude the possibility that other brain regions, such as the amygdala, might contribute to the observed behavioral phenotype. Therefore, additional investigations of gene expression in the amygdala under the experimental conditions should be performed.

In summary, we demonstrated, for the first time, the possibility that a neuropeptide, CGRP, can reduce fear memory retention through epigenetic regulation, via the PKD-HDAC5-Npas4 pathway. These results suggested that CGRP induces fear memory retention and support the view that Npas4 can contribute to quickly erasing memories or perhaps eliminate their creation. This study may improve our understanding of CGRP functions during fear memory formation. These results form the basis of potential treatment options for post-traumatic stress disorder (PTSD). If CGRP can be administered specifically to the brain post-trauma, the suppression of damaging fear memory formation associated with PTSD may be possible. CGRP treatment could represent a component of emergency PTSD treatments.

## Materials and methods

### Animals

All animal procedures were performed as previously described^6,47,48^, in accordance with the ARRIVE guidelines and U.S. National Institutes of Health (NIH) Guide for the Care and Use of Laboratory Animals (NIH Publication No. 80-23, revised in 1996). All experiments were approved by the Animal Care and Use Committee of the Okayama University of Science. According to these guidelines, efforts were made to minimize the number of animals used and their suffering. We purchased C57BL/6J male mice from Shimizu Experimental Animals (Shizuoka, Japan). A total of 247 mice were used in this experiment. All animals were housed in the Animal Research Center of Okayama University of Science, at a controlled ambient temperature of 22 °C, with 50 ± 10% relative humidity, and a 12 h light/dark cycle (lights on at 7:00 AM). Animals were group-housed, and each home cage contained five to six mice.

### Behavioral assessments

#### Open field test

Open field test was performed as previously described^6,47,48^. We used 6 mice to saline and 7 mice to CGRP i.c.v. administration. Mice were placed in the center of a circular open field chamber (57.5 cm diameter, 32 cm high). The floor was divided into 19 sections, with each section having nearly the same area. The center area was defined by a circle with a 35.5 cm radius (3957 cm^2^). Locomotor activity was scored in terms of line crossings when a mouse removed all four paws from one section and entered another. All animal behaviors were videotaped using a digital camera. Line crossings, rearing activity, and the time spent in the center area were measured over the course of 3 min, using a stopwatch and a counter.

#### Morris water maze test

Spatial learning and memory were assessed using the MWM task and performed as previously described^49^. We used 8 mice to each group. We measured the latency to reach the platform for each mouse. Mice were allowed to explore the platform location for 90 s. The experimental trials occurred over 4 days. The day after the last training trial, mice were given CGRP injection into the brain and we performed a probe test^49^. The ratio between the time spent in the target quadrant (where the platform was previously located) and the time spent in the opposite quadrant (% of time spent) was used as an index of retrieval memory.

#### Y-maze test

The Y-maze used in this study was performed as previously described^50^. We used 8 mice to each group. Mice were injected with CGRP or saline and then returned to their home cages. After 24 h, the mice were placed at the end of one arm of the Y-maze and allowed to freely explore the maze for 8 min. An alternation is defined as the entry into all three arms consecutively, and the number of maximum spontaneous alternations was calculated as the total number of arms entered, minus two. The percentage of alternation was calculated as (actual alternations/maximum alternations) × 100.

#### Passive avoidance test

Passive avoidance test was performed as previously described^49^. We used 8 mice to each group. On the first day, each subject was allowed to enter the dark chamber for 90 s, to adapt to the apparatus. On day 2, one more trial was performed, and immediately after the second trial, the animals received an electric foot shock when they entered the dark chamber. The mice were placed back in the home cage and received CGRP (0.5 nmol) or saline. On day 3, the step-through latencies were measured 24 h after treatment. The latency to enter the dark box was recorded, up to 300 s.

#### Contextual fear conditioning test

A total of 136 mice were used in this experiment. Mice were placed in a round chamber apparatus (diameter: 16.5 cm; height: 7.5 cm). The training sessions consisted of a 120-s exploration period, followed by 4 foot-shocks (training) (foot shock intensity: 0.3 mA, 2-s duration). Mice were injected with CGRP or saline 1 h after receiving the foot shocks and were then returned to their home cages. Freezing behavior was defined as the absence of movement, except for respiration, 24 h after training. Chambers were cleaned using 70% ethanol between each trial. In the present study, we focused on the effects of CGRP on short-term memory, within the first 24 h after fear exposure. Although 1 or 3 h after CGRP administration might provide a more robust outcome with regard to the levels of mRNA or protein expression, our primary goal was to observed behavioral changes after fear exposure.

#### RNA extraction

Total RNA was extracted from the mice hippocampus, placed in RNAlater (Life Technologies Co., Tokyo, Japan), and stored at − 30 °C. Total RNA was extracted using the RNeasy Plus Micro kit (Qiagen, Tokyo, Japan) and mRNA extraction were performed as previously described^6,47,48^.

#### Quantitative analysis by real-time PCR

The reverse-transcribed mixture was used as a template for subsequent real-time PCR assays. Real-time PCR was performed as previously described^6,47,48^. The data were analyzed using the mean threshold cycle equation. The primer information is shown in Table 1. Actin (*Actin*) served as an internal control. The threshold cycle values for both the target (*Npas4*) and internal control (*Actin*) were determined. The fold change of each gene was normalized to that of *Actin* and was calculated for each sample, relative to the expression levels in the control samples. The specificity of amplification was verified by the monophasic characteristic of the melting curve generated for each amplification product by the Eco Real-Time PCR System (Illumina Inc., Tokyo, Japan) at the end of the PCR.

**Table 1:** Oligonucleotide sequences for real-time PCR amplification.

	Forward	Reverse
*Npas4*	5ʹ-CATGCTAAGGACCTAGCCCTACTG-3ʹ	5ʹ-GGTGTAGCAGTCCATACCATGA-3ʹ
*Actin*	5ʹ-GGTCAGAAGGACTCCTATGTG-3ʹ	5ʹ-GGTGTGGTGCCAGATCTTCTCC-3ʹ

#### Western blotting

For western blot analyses, we conducted as previously described^47^. The collected hippocampus were placed in RNAlater (Life Technologies) and homogenized in a sodium dodecyl sulfate (SDS) sample buffer. Protein extracts were separated by SDS–polyacrylamide gel electrophoresis and then transferred onto a polyvinylidene difluoride membrane (HybondP; GE Healthcare UK Ltd.). The membrane was blocked with a blocking agent (GE Healthcare) and then incubated at 4 °C overnight with the following primary antibodies: mouse monoclonal anti-Npas4 (1:5000, Santa Cruz Biotechnology, Inc.), rabbit polyclonal anti-HDAC5 (phospho S498) (1:5000, Abcam plc, Cambridge, UK), mouse monoclonal anti-HDAC5 (1:5000, Santa Cruz Biotechnology), and rabbit polyclonal anti-PKD1/2/3 PKC micro antibody (Gene Tex, Inc. CA). After washing with tris-buffered saline containing 0.1% (v/v) Tween 20, the membranes were incubated with horseradish peroxidase-conjugated secondary antibody (1:20,000) for 1 h at room temperature. The antibody-reactive bands were visualized using a chemiluminescent substrate kit (GE Healthcare). Bands were analyzed by densitometry, using ImageJ (https://imagej.nih.gov/ij/), and the glyceraldehyde 3-phosphate dehydrogenase (GAPDH) contents, which were detected using a rabbit anti-GAPDH antibody (1:20,000; Sigma-Aldrich CO. LLC. Japan), were used to ensure that the same amount of protein was loaded in each lane.

#### Drug treatments

##### Intracerebroventricular (i. c. v.) administration

Rat CGRP (Sigma-Aldrich; 0.5 nmol) and CGRP_8-37_ (Sigma-Aldrich; 0.5 nmol) were diluted in saline. Isoflurane (1.5–2.0%) was used for brief anesthesia during i.c.v. injections. Drug administration was performed by the direct injection into the right lateral ventricle, through the intact scalp, aiming at 1 mm posterior to bregma and 1 mm right from the midline, as described previously^51^. H89 (1 mg/kg, Sigma-Aldrich) was administered by intraperitoneal injection, 30 min before CGRP injection.

##### Chromatin immunoprecipitation (ChIP)

A total of 50 mice were used in this experiment. CGRP or saline was administered i.c.v. for 24 h before cross-linking. The mice were deeply anesthetized with three types of mixed anesthetic agents^52^, medetomidine hydrochloride (Domitol, Meiji Seika Pharma Co., Ltd., Tokyo, Japan, 0.3 mg/kg), midazolam (Dormicum, Astellas Pharma Inc., Tokyo, Japan, 4.0 mg/kg), and butorphanol (Vetorphale, Meiji Seika Pharma Co., Ltd., 5.0 mg/kg), administered intraperitoneally and perfused transcardially with saline, followed by 1% paraformaldehyde, pH 7.4. The hippocampus was post-fixed for 10 min in 1% paraformaldehyde, and then glycine (330 mM) was added. The chromatin was then sheared into approximately 0.5–1-kb fragments and immunoprecipitated using an anti-HDAC5 antibody (1:100 for the hippocampal sample, Santa Cruz Biotechnology), acetylated Histone3 antibody (1:100, Abcam plc) or Histone3 antibody (1:100, Abcam plc). Immunoprecipitated DNA fragments were amplified and quantitated by qPCR (Eco Real-Time PCR System (Illumina Inc.), using PCR primers specific for *Npas4*, which were designed around the putative enhancer regions of *Npas4* (Table 2).

**Table 2:** Oligonucleotide sequences for ChIP.

	Forward	Reverse
*Npas4*	5ʹ-TGCCAGGCTATTTTTGGTTC-3ʹ	5ʹ-CTGTATGCCCCCAATGTCTC-3ʹ

##### siRNA constructs

The short interfering RNA (siRNA) reagents used were Dharmacon’s Accell siRNA, SMARTpool (Accell Mouse Npas4 [225872] siRNA-SMART pool, 10 nmol or Accell Prkd1 siRNA, 10 nmol) and Non-targeting siRNA (GE Healthcare). The mice were divided into four groups: saline with non-targeting control, saline with *Npas4* or *Prkd1*-siRNA, CGRP with non-targeting control, and CGRP with *Npas4* or *Prkd1*-siRNA. Non-targeting control and *Npas4* or *Prkd1*-siRNA were administered via i.c.v. injection, 24 h prior to fear conditioning on day 1. After fear conditioning, mice were administered saline or CGRP, on day 2. Isoflurane was used for brief anesthesia during i.c.v. injections. Freezing behavior was evaluated on day 3.

### Statistical analysis

All data are expressed as the mean ± standard error of the mean (S.E.M). GraphPad Prism 7 software (GraphPad Software Inc., San Diego, CA, USA) was used for all statistical analyses. Comparisons between two values were analyzed using the Welch’s t-test. An analysis of variance (ANOVA), followed by Tukey’s multiple comparison test, was used to determine significant differences, where appropriate. Two-way ANOVAs were also performed. A *p* value < 0.05 was considered significant.

## Supplementary Information

Supplementary Information
