# Supplementary material for: Npas4 impairs fear memory via phosphorylated HDAC5 induced by CGRP administration in mice
Source: Sci Rep. 2021 Mar 26;11:7006. doi: 10.1038/s41598-021-86556-w (PMC7997869; doi:10.1038/s41598-021-86556-w)
Supplement: Supplementary file 1 — Supplementary Information [file 41598_2021_86556_MOESM1_ESM.docx]

**Title:** Npas4 impairs fear memory via phosphorylated HDAC5 induced by CGRP administration in mice

**Authors:** Narumi Hashikawa-Hobara^1^*, Shuta Mishima^1^, Chihiro Okujima^1^, Youdai Shitanishi^1^, Naoya Hashikawa^1^

**Affiliations:**

^1^ Department of Life Science, Okayama University of Science, 1-1 Ridai-cho, Kita-ku, Okayama 700-0005, Japan.

*To whom correspondence should be addressed: Narumi Hashikawa-Hobara, 1 Ph.D.

^1^Department of Life Science, Okayama University of Science, 1-1 Ridai-cho, Kita-ku Okayama 700-0005, Japan

+81-86-256-9719

E-mail: [hobara@dls.ous.ac.jp](mailto:hobara@dls.ous.ac.jp)

All authors’ contact information

Narumi Hashikawa-Hobara: [hobara@dls.ous.ac.jp](mailto:hobara@dls.ous.ac.jp)

Shuta Mishima: [s17lm12ms@ous.jp](mailto:s17lm12ms@ous.jp)

Chihiro Okujima: [s20lm04oc@ous.jp](mailto:s20lm04oc@ous.jp)

Youdai Shitanishi: [s20lm06sy@pus.jp](mailto:s20lm06sy@pus.jp)

Naoya Hashikawa: [hashikawa@dls.ous.ac.jp](mailto:hashikawa@dls.ous.ac.jp)

**Supplementary Materials**


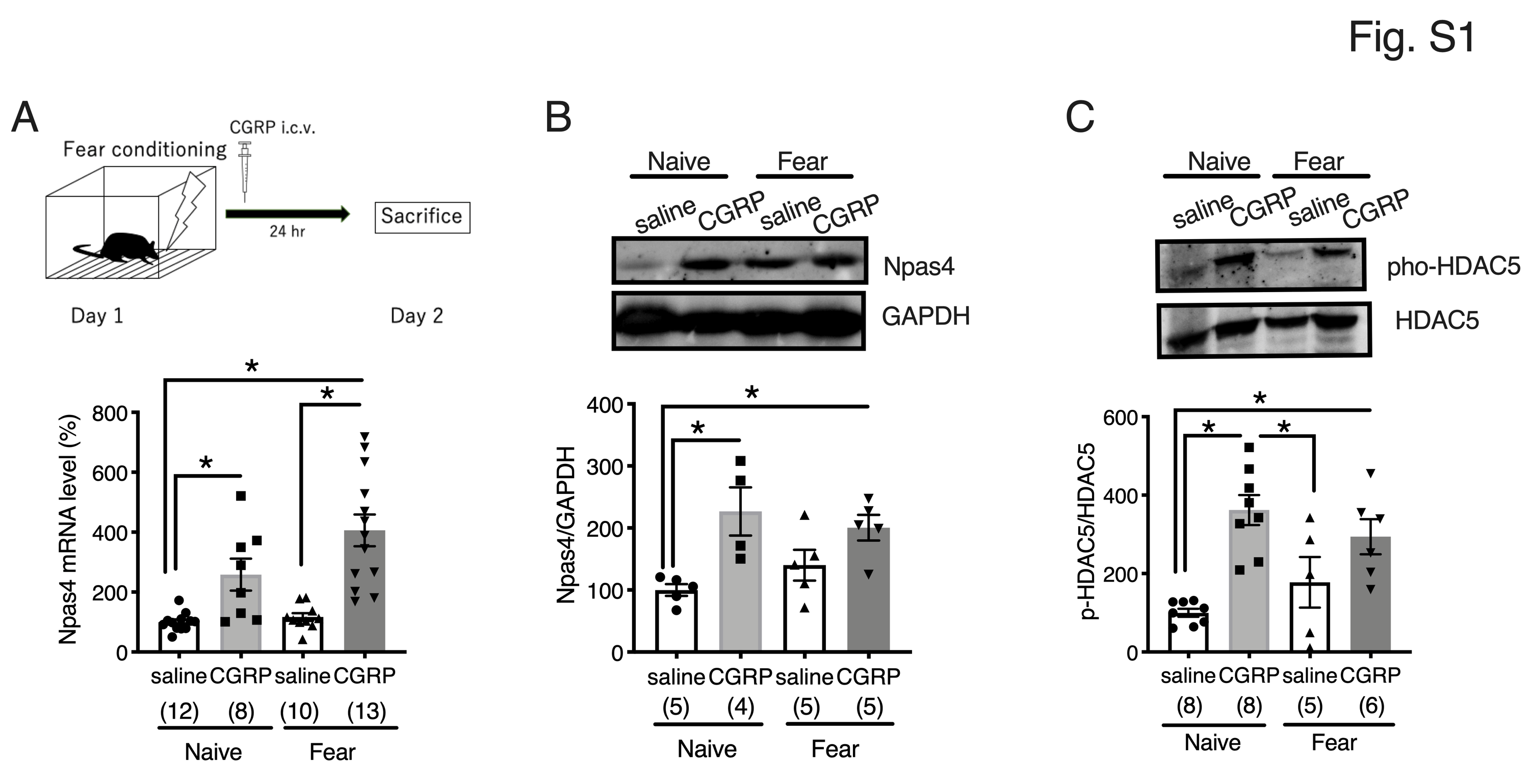


Supplementary Figure 1,

**Fig. S1.** Npas4 or phosphorylated HDAC5 levels in saline or CGRP treatment between naïve controls and 24 hr after fear conditioning. (**A**) Npas4 mRNA (One-way ANOVA with Tukey’s test F_3, 39_ = 15.41). (**B**) Npas4 protein (One-way ANOVA with Tukey’s test F_3, 15_ = 5.608). (**C**) Phosphorylated HDAC5 expression (One-way ANOVA with Tukey’s test F_3, 23_ = 10.32). Each bar indicates the mean ± S.E.M. * p < 0.05. Numbers in parentheses indicate the animal numbers for each group.


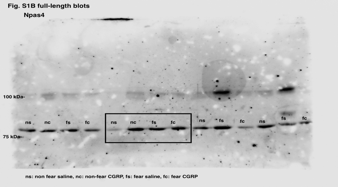


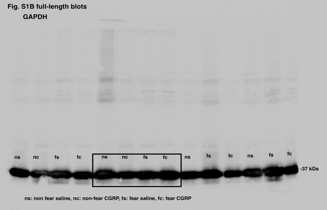


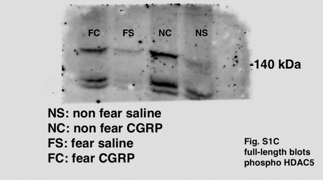


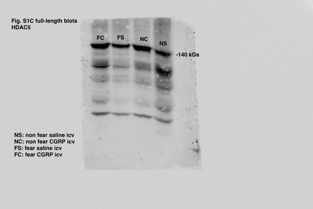


The full-length blots of Figure S1B and S1C are presented.

Supplementary Figure 2,


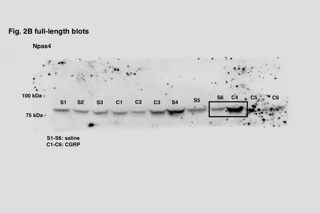

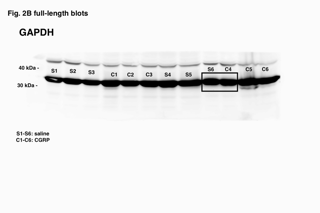


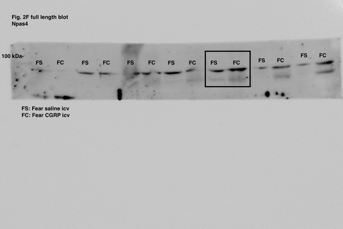

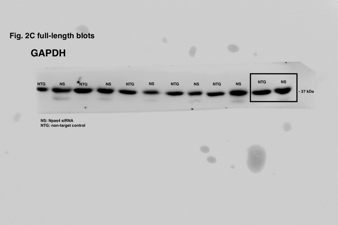

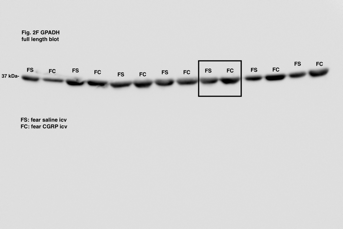

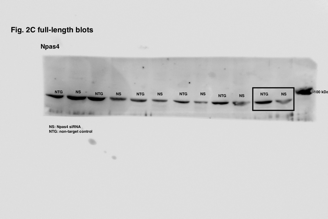


Figure S2. The full-length blots of Figure 2B, 2C and 2F are presented.

Supplementary Figure 3


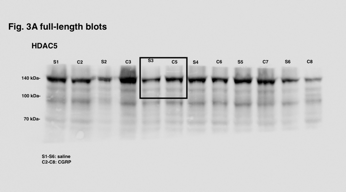

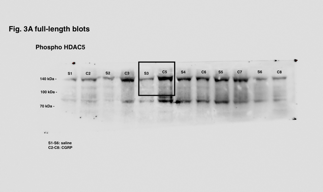


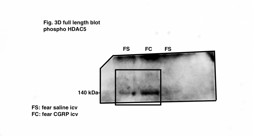


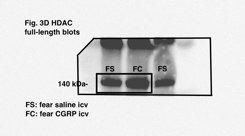


Figure S3. The full-length blots of Figure 3A and 3D are presented.


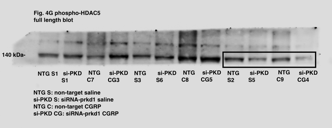

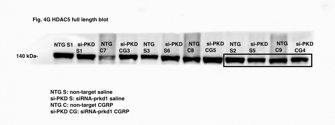

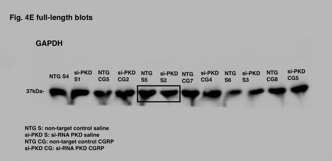

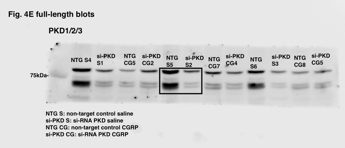

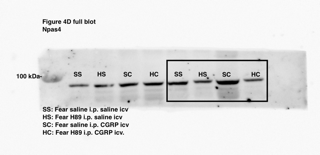

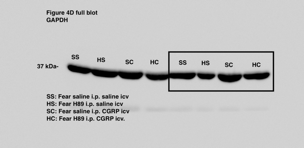

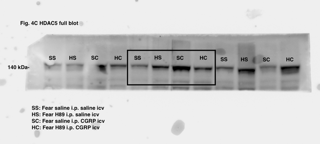

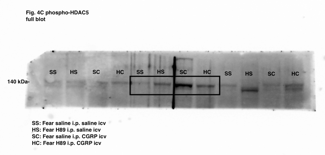

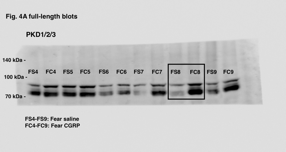

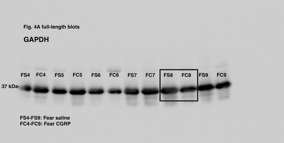
Supplementary Figure 4


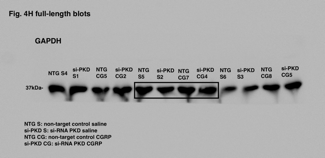

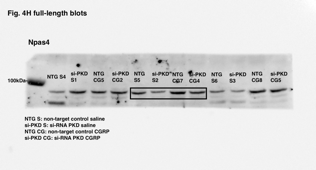


Figure S4. The full-length blots of Figure 4A, 4C, 4D, 4E, 4G and 4H are presented.
